# Determining vector competence of *Aedes aegypti* from Ghana in transmitting dengue virus serotypes 1 and 2

**DOI:** 10.1186/s13071-021-04728-z

**Published:** 2021-04-29

**Authors:** Michael Amoa-Bosompem, Daisuke Kobayashi, Kentaro Itokawa, Katsunori Murota, Astri Nur Faizah, Faustus Akankperiwen Azerigyik, Takaya Hayashi, Mitsuko Ohashi, Joseph H. Kofi Bonney, Samuel Dadzie, Cuong Chi Tran, Phong Vu Tran, Ryosuke Fujita, Yoshihide Maekawa, Shinji Kasai, Shoji Yamaoka, Nobuo Ohta, Kyoko Sawabe, Shiroh Iwanaga, Haruhiko Isawa

**Affiliations:** 1 Department of Environmental Parasitology, Tokyo Medical and Dental University, Bunkyo, Tokyo, Japan; 2 Department of Medical Entomology, National Institute of Infectious Diseases, Shinjuku, Tokyo, Japan; 3 Department of Parasitology, Noguchi Memorial Institute for Medical Research, University of Ghana, College of Health Sciences, Legon, Accra, Ghana; 4 Pathogen Genomics Center, National Institute of Infectious Diseases, Shinjuku, Tokyo, Japan; 5 Graduate School of Agricultural and Life Science, The University of Tokyo, Bunkyo, Tokyo, Japan; 6 Department of Virology, Noguchi Memorial Institute for Medical Research, University of Ghana, College of Health Sciences, Legon, Accra, Ghana; 7 Department of Molecular Virology, Tokyo Medical and Dental University, Bunkyo, Tokyo, Japan; 8 Medical Entomology and Zoology Department, National Institute of Hygiene and Epidemiology, Hanoi, Vietnam; 9 Laboratory of Sanitary Entomology, Faculty of Agriculture, Kyushu University, Fukuoka, Japan; 10 Faculty of Health Science, Suzuka University of Medical Science, Suzuka, Mie Japan; 11 Present Address: Laboratory of Sanitary Entomology, Faculty of Agriculture, Kyushu University, Fukuoka, Japan; 12 Present Address: Kyushu Research Station, National Institute of Animal Health, NARO, Chuzan, Kagoshima, Japan; 13 Present Address: Department of Molecular Protozoology, Research Center for Infectious Disease Control, Research Institute for Microbial Diseases, Osaka University, Suita, Osaka Japan

**Keywords:** *Aedes aegypti*, Vector competence, Dengue virus, Susceptibility, Ghana, West Africa

## Abstract

**Background:**

Dengue virus (DENV) is a mosquito-borne arbovirus transmitted by *Aedes* mosquitoes, but is not endemic in all areas where this vector is found. For example, the relatively sparse distribution of cases in West Africa is generally attributed to the refractory nature of West African *Aedes aegypti* (*Ae. aegypti*) to DENV infection, and particularly the forest-dwelling *Ae*. *aegypti formosus*. However, recent studies have shown these mosquitoes to be competent vectors within some West African countries that have suffered outbreaks in the past, such as Senegal. There is however little information on the vector competence of the *Ae. aegypti* in West African countries such as Ghana with no reported outbreaks.

**Methods:**

This study examined the vector competence of 4 *Ae. aegypti* colonies from urban, semi-urban, and two rural locations in Ghana in transmitting DENV serotypes 1 and 2, using a single colony from Vietnam as control. Midgut infection and virus dissemination were determined by quantitative reverse transcription polymerase chain reaction (qRT-PCR), while the presence and concentration of DENV in the saliva of infectious mosquitoes was determined by the focus forming assay.

**Results:**

There were significant differences in the colonies’ susceptibility to virus infection, dissemination, and transmission. All examined Ghanaian mosquitoes were refractory to infection by DENV serotype 2, while some colonies exhibited potential to transmit DENV serotype 1. None of the tested colonies were as competent as the control group colony.

**Conclusions:**

These findings give insight into the possible risk of outbreaks, particularly in the urban areas in the south of Ghana, and highlight the need for continuous surveillance to determine the transmission status and outbreak risk. This study also highlights the need to prevent importation of different DENV strains and potential invasion of new highly vector-competent *Ae. aegypti* strains, particularly around the ports of entry.

**Graphic Abstract:**

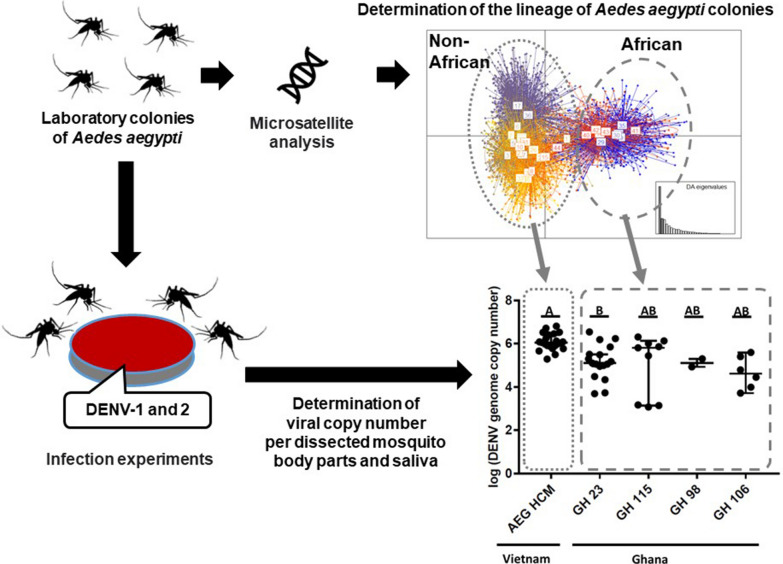

## Background

Dengue fever is among the most relevant vector-borne diseases, causing an estimated 390 million infections and 25,000 deaths per year [1]. The distribution of dengue fever cases continues to grow; several countries in Europe and Africa have recently reported cases for the first time—imported or possibly locally transmitted— while other countries like Japan are experiencing outbreaks after a period of > 70 years with no reported cases [2–6]. This progressive spread of dengue fever into new areas and its reappearance in previously endemic areas makes it currently one of the most important arboviral diseases.

Dengue fever is caused by the flavivirus dengue virus (DENV), which consists of four serotypes (DENV 1–4) that are phylogenetically and antigenically distinct [7–9]. The primary vector for all serotypes is the mosquito *Aedes aegypti* (*Ae. aegypti*). Although these four serotypes have all been reported in Africa, Asia, and the Americas, there are significant differences in the incidence and intensity of outbreaks in these geographical areas. For example, the continents of Asia and Americas typically report over a million total infections annually. Africa, on the other hand, typically records hundreds of cases, which may increase to thousands in a severe outbreak, but still significantly lower than those of Asia and the Americas [7, 10]. These apparent differences in outbreak incidence and intensity have been attributed to many factors, including the role of the *Aedes* vector.

*Ae. aegypti* is the most medically significant invasive mosquito species, and serves as the primary vector of DENV, yellow fever virus, Zika virus, and chikungunya virus [11, 12]. *Ae. aegypti* mosquitoes are historically divided into three subspecies [11], but only *Ae. aegypti aegypti* and *Ae. aegypti formosus* are recognized as subspecies of *Ae. aegypti s.l.* [13]. These two subspecies were originally distinguished from each other based on morphological features, such as scaling pattern, as well as their geographic distribution and host preference. *Ae. aegypti aegypti* was described as the domestic subspecies distributed across tropical and subtropical areas, with light tan coloring, pale scales on the first (or both) abdomen tergite(s), and a preference for human blood, while *Ae. aegypti formosus* was considered the ancestral subspecies, dark black in coloration, with no scales on the first abdominal tergite, and restricted to sub-Saharan Africa in undisturbed forests, with a preference for nonhuman blood [11, 13]. However, subsequent reports pointed out the high variability of scale patterns within the *Ae. aegypti s.l.* population, making subspecies classification via morphology increasingly difficult, particularly in West Africa where white scales have been observed on some black *Ae. aegypti* [11, 13, 14]. Furthermore, breeding site and host preference for classification also has limitations in West Africa where black *Ae. aegypti* lacking white scales are reportedly domesticated and anthropophilic (i.e. prefer human blood meals) [14].

These limitations have necessitated the use of molecular techniques to complement morphological identification. Although this approach has proven effective in East Africa where there is a clear correlation between genotypic microsatellite differences, morphology, and distinct behavioral patterns, this is not the case in West Africa, where reports suggest that no correlation exists between such genetic markers and morphology or behavioral patterns [13, 14]. Furthermore, molecular studies indicate distinct genetic differences between East African *Ae. aegypti aegypti* and *Ae.aegypti formosus* and the West African *Ae. aegypti s.l.* [13]. This study therefore employed the method reported by Dickson et al. [13] by referring to collected *Ae. aegypti* based on their breeding site, habitat, and phytogeographic region.

With respect to DENV vector competence, studies have been carried out within West Africa in Nigeria and Senegal where DENV outbreaks have been reported in the past. Varying degrees of competence were reported contrary to earlier studies suggesting *Ae. aegypti* in West Africa were not competent vectors [13–16]. In a study in Senegal, for example, classic *Ae. aegypti formosus*—forest dwelling, dark black with no white scales on the first abdominal tergite–had an 80% susceptibility rate to DENV-2, with 60% infection dissemination [13]. Furthermore, although a study comparing *Ae. aegypti* strains from Puerto Rico and Nigeria reported relatively lower susceptibility of the Nigerian strain to DENV-2 infection, 25% of tested mosquitoes were susceptible to infection at a 14% dissemination rate [15]. Although these studies have provided valuable information on *Ae. aegypti* competence as vectors of DENV in West Africa, the transmission potential of these strains was not determined via measurement of the presence and concentration of the virus within mosquito saliva [17]. Furthermore, similar studies have not yet been conducted in West African countries such as Ghana, with no reported DENV outbreaks, despite an abundance of the *Ae. aegypti* vector [18].

Ghana is a West African country, north of the Equator, and shares borders with Burkina Faso, Gulf of Guinea, Cote d’Ivoire, and Togo in the north, south, west, and east, respectively. Ghana has a warm tropical climate with an abundance of *Ae. aegypti* mosquitoes [18]. Cote d’Ivoire and Burkina Faso reported DENV outbreaks in 2015 and 2016, respectively [2, 19]. Although there have been no documented outbreaks in Ghana, there was a report containing serological evidence of potential DENV exposure among children positive for malaria parasites in 2015 [20]. However, that study was limited in that the IgM Capture DxSelect antibody capture kit used is cross-reactive with other flaviviruses, including yellow fever virus [21], which is endemic in Ghana but currently under control due to active vaccination. Nonetheless, DENV infection was confirmed in Ghana in two children and four adults in two separate studies in 2018 [2, 19], suggesting possible local transmission. It has therefore become increasingly important to confirm the competence of *Ae. aegypti* in Ghana as an assessment of the risk of DENV transmission or outbreak.

The vector competence of four *Ae*. *aegypti* colonies collected from distinct locations in Ghana (i.e. two southern, two northern) [18] was herein determined for transmission of DENV in terms of infection, dissemination, and transmission rates. An *Ae*. *aegypti aegypti* colony collected from Ho Chi Minh City, Vietnam was used as a control for comparative analysis.

## Methods

### Mosquito collection

*Aedes aegypti* larvae were collected from four locations in Ghana in 2016 [18] (Table 1). Collection sites included artificial containers in domestic areas of rural villages, which are tourist attraction sites, as well as used tyres in urban, semi-urban, and rural villages (Table 1). The collected larvae were transported to the Noguchi Memorial Institute for Medical Research (NMIMR), reared to adults, and blood fed for oviposition. The eggs were subsequently transported to the National Institute of Infectious Diseases (NIID) in Japan and used to establish four distinct *Ae. aegypti* colonies. Similarly, *Ae. aegypti aegypti* larvae collected from used tyres in Ho Chi Minh City were reared to adults and allowed to lay eggs, which were then transported to NIID to establish the control group. The first three generations were used for stabilizing and expanding the colonies, after which the fourth to ninth generations were used for infection experiments as described below. Adult mosquitoes were sustained on sugar meals and maintained at 25 °C and 70% humidity with a 16 h light/8 h dark cycle. Eggs collected on filter paper were dried and stored for up to three months.

**Table 1:** List of *Ae. aegypti* colonies used for DENV infection experiments

Colony name	Larval collection site	Breeding site	Habitat	Phytogeographic region	Latitude and longitude (°)	Country	Collection date
GH 23	Hohoe	Tyre	Semi-urban	Semi-deciduous forest	7.1655, 0.4783	Ghana	August 2016
GH 115	Accra	Tyre	Urban	Grassland-Savannah	5.601130, −0.236980		
GH 98	Larabanga	Artificial container	Rural Village	Woodland-Savannah	9.219346, −1.860639		
GH 106	Jirapa	Tyre	Rural village	Woodland-Savannah	10.53645, −2.70393		
AEG HCM	Ho Chi Minh City	Tyre	Urban	Tropical Savannah	10.8, 106.7	Vietnam	September 2016

### Population assignment of *Ae. aegypti* colonies

The origin(s) of the colonies was confirmed by genotyping 12 microsatellite loci [22] of individuals of the fifth generation (F_5_) using methods previously described [23]. Briefly, genomic DNA was singly extracted from 10 mosquitoes per colony, five males and five females, using the MagExtractor-Genome kit (TOYOBO, Osaka, Japan). Microsatellite markers and primers, dinucleotide and trinucleotide repeats, developed by Slotman et al. [24] and Brown et al. [22] and the Type-it Microsatellite PCR Kit (Qiagen, Hilden, Germany) were used in the polymerase chain reaction (PCR). Multiplex pairings and primer/fluorophore combinations were performed as previously described [25]. The purified PCR product was electrophoresed in the presence of the 500 LIZ size standard (GeneScan, Thermo Fisher Scientific, Waltham, MA) on an ABI3130 sequencer. Analysis of genotypic data was performed by discriminant analysis of principal components (DAPC) using the Adegenet package in R 3.6 [26, 27].

### DENV preparation

Two serotypes of DENV, with low passage numbers, were used in this study. Both serotypes were isolated from patient blood: DENV-1 (strain D1/Hu/Saitama/NIID100/2014) from a patient in Japan [5] and DENV-2 (strain DENV-2/GH/NMIMR-BC-UG-F299/2017) from a patient in Ghana [2]. DENV-1-positive serum was first inoculated on FcγR-expressing baby hamster kidney (BHK) cells (Department of Virology I, NIID, Japan), after which the culture supernatant was inoculated on Vero cells (Department of Veterinary Science, NIID, Japan) and subsequently inoculated on C6/36 cells (European Collection of Authenticated Cell Cultures). The resulting culture supernatant was stored at −80 °C until used. DENV-2 positive serum on the other hand was inoculated on C6/36 cells and the resulting culture supernatant passaged twice on C6/36 cells and stored at −80 °C until used. The fourth passage of both DENV serotypes in Vero cells was used for all experiments.

Prior to the infection experiment, growth curves were performed for both serotypes to determine the optimal time between virus inoculation and harvesting for infection. DENV-1 or DENV-2 was inoculated on Vero cells with a multiplicity of Infection (MOI) value of 0.1. DENV-1 and DENV-2 were propagated for 6 and 5 days, respectively, to an average titer of about 2 × 10^6^ focus forming units/ml (FFU/ml). Harvested culture-supernatant was centrifuged at 180×*g* for 10 min, pellets discarded, and stored on ice until used [28]. The supernatant was subsequently mixed with defibrinated rabbit blood (Nippon Bio-Supp. Centre, Tokyo, Japan) laced with 2 mM adenosine triphosphate (ATP) at a ratio of 1:1, for a resulting titer of approximately 1 × 10^6^ FFU/ml for mosquito infection. Viral titer was confirmed in each experiment.

### Mosquito infection with DENV

Female mosquitoes, 7–10 days, old were starved overnight and subsequently fed on infectious blood using the Hemotek™ 5W1 membrane feeding system for blood-sucking insects (Hemotek Ltd, Blackburn, UK). Briefly, 3 ml of infectious blood was pipetted into the reservoir and covered with a membrane of swine intestine. The reservoir was then applied to the FU1 feeder (Hemotek Ltd) and the blood allowed to warm up for about 1 min. The FU1 feeder containing the infectious blood meal was then placed on the collection cups for 1 h to allow for blood feeding.

After feeding, CO_2_ was used to anesthetize the mosquitoes after which they were divided, on ice, into two groups: fully engorged or unfed/partially engorged mosquitoes. Fully fed mosquitoes were randomly divided into two cages containing an oviposition tray and a 3% sugar meal and maintained at 28 °C with a 16 h light/8 h dark cycle, one cage for 7 days and the other for 14 days. Unfed and partially fed mosquitoes were excluded from the study. Only mosquitoes that were alive at the time of collection were subjected to further analysis.

### Salivation and mosquito dissection

Mosquitoes harvested at 7 or 14 days post infection (dpi) were CO_2_-anesthetized and immobilized by removing their wings and legs. The proboscises of immobilized mosquitoes were inserted into a 10-µl pipette tip containing 5 µl heat-inactivated fetal bovine serum (FBS, Biowest, Nuaillé, France) and allowed to salivate for 1 h. After 1 h, the saliva was added to 495 µl of Eagle’s minimum essential medium (MEM, Sigma-Aldrich, St. Louis, MO) supplemented with 2% FBS, 200 U/ml penicillin (Thermo Fisher Scientific), 200 µg/ml streptomycin (Thermo Fisher Scientific), and 5 µg/ml amphotericin B (Thermo Fisher Scientific).

After salivation, the mosquitoes were dissected on ice into two groups: thorax/abdomen and head/wings/legs. All samples were stored at −80 °C until used.

### DENV detection and quantification in the mosquitoes

Each mosquito was screened for DENV infection, dissemination, and transmission. The thorax/abdomen was screened first for infection, and if positive, then the head/wings/legs were screened for dissemination. Saliva samples of mosquitoes exhibiting both infection and dissemination was screened to determine transmission potential. Quantification of DENV in the thorax/abdomen and head/wings/legs was performed by quantitative real-time polymerase chain reaction (qRT-PCR) while DENV in the saliva was quantified by the focus forming assay. The focus forming assay was performed with the mAb4G2-antibody produced in mouse hybridoma cells, D1-4G2-4–15 (ATCC HB-112, American Type Culture Collection), and the Dako HRP Labelled Polymer Anti-mouse, and Liquid DAB+ Substrate Chromogen System (Agilent Technologies, Santa Clara, CA) [18].

In preparing for qRT-PCR, gene-specific primers for the two DENV serotypes were designed using GENETYX version 13 software (Genetyx Corp., Tokyo, Japan), while the probes were synthesized by Integrated DNA Technologies (Table 2). The target region for each set of primers and probes was amplified and transcribed in vitro using the T7 RNA polymerase. The transcribed RNA was treated with DNAse and purified using the RNeasy MinElute Cleanup Kit (Qiagen, Hilden, Germany); RNA concentration was determined using a Qubit fluorometer (Thermo Fisher Scientific) and combined with its molecular weight to calculate the RNA copy number. For each qRT-PCR reaction, 100-fold serial dilutions of the RNA from 10^0^ to 10^10^ copies were used as standards. Each qRT-PCR reaction also included a negative control containing no RNA copy.

**Table 2:** Primer and probe sequences used in the qRT-PCR analysis

Virus	Primer name	Primer sequence (5′–3′)
DENV-1	D1MGBEn469s	GAACATGGRACAAYTGCAACYAT
	D1MGBEn536r	CCGTAGTCDGTCAGCTGTATTTCA
	D1MGBEn493p (probe)	ACACCTCAAGCTCC
DENV-2	D2MGBEn493s	ACACCACAGAGTTCCATTACAGA
	D2MGBEn568r	CATCTCATTGAAGTCNAGGCC
	D2MGBEn545p (probe)	CGATGGARTGCTCTC

RNA was extracted using the Nucleospin RNA extraction kit (Macherey–Nagel, Dueren, Germany) following the manufacturer’s protocol. Extracted RNA was subjected to qRT-PCR using the TaqMan Fast Virus 1-Step Master Mix (Thermo Fisher Scientific) on the PikoReal 96 real-time PCR system (Thermo Fisher Scientific) [29]. Each reaction mix had 1× TaqMan Virus 1-step Master Mix, 900 nM of both the forward and reverse primers, 250 nM probe, and 1 µl of RNA in a 10 µl reaction volume. The reaction mix was pre-incubated at 50 °C for 5 min, followed by denaturation at 95 °C for 20 s and 40 cycles of 95 °C for 3 s and 60 °C for 30 s. Each sample was run in triplicate. Analysis of qRT-PCR data was conducted using PikoReal version 2.2 software 2.2 (Thermo Fisher Scientific). Samples were considered negative when the quantification cycle (Cq) value was 35 and above.

### Data analysis and statistics

Data and statistical analyses were carried out using GraphPad Prism version 7.00 software for Microsoft Windows. Statistical analysis of viral titers and viral genome number was performed by the Kruskal–Wallis test with Bonferroni correction. The chi-square test with Bonferroni correction was used to determine the significant differences in the proportion of mosquitoes harboring midgut infection, disseminated infection, and infectious saliva.

## Results

### Population assignment of *Ae. aegypti* colonies

**Fig. 1:**
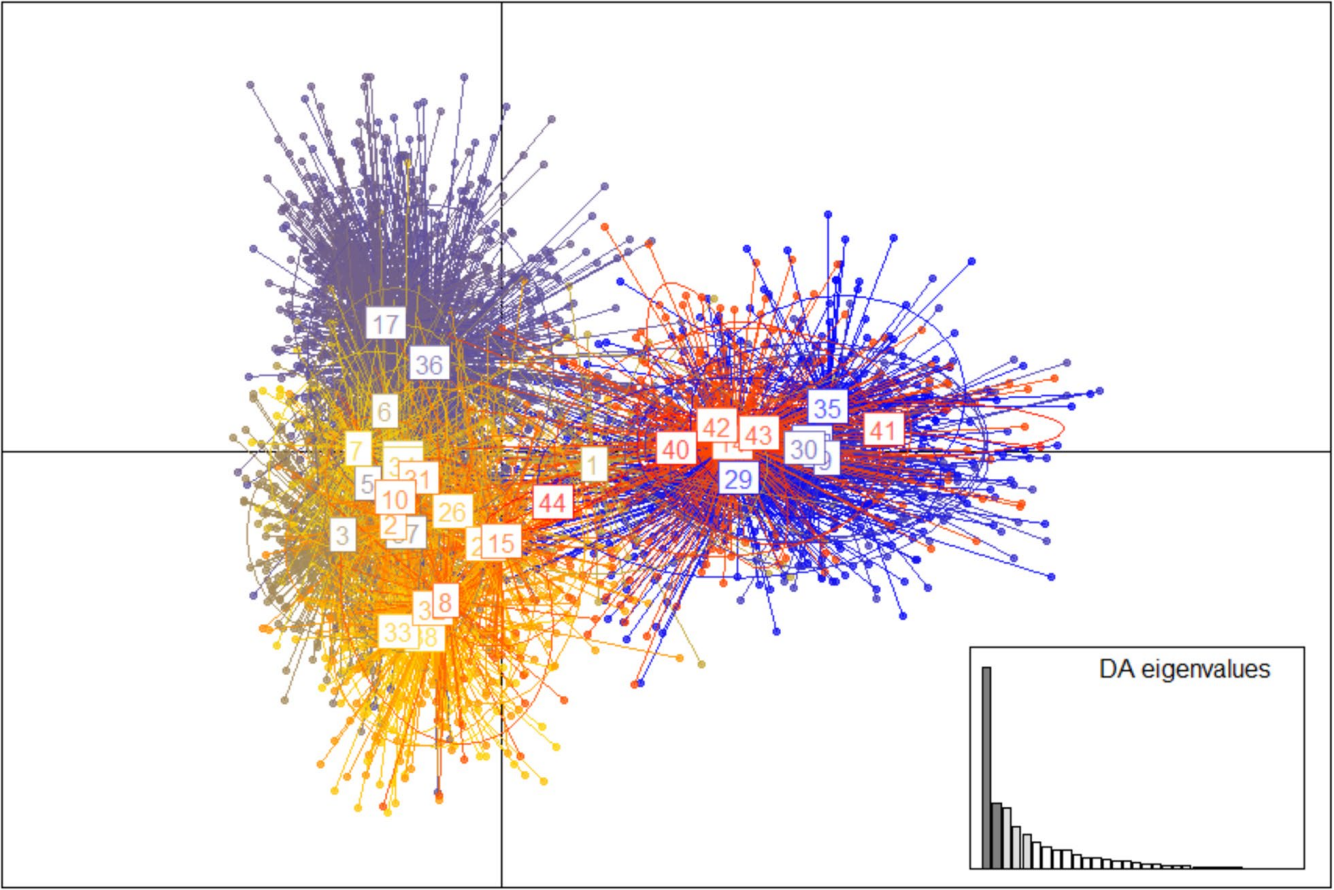
Lineage of *Ae. aegypti* colonies used in this study. Results of discriminant analysis of principal components comparing 12 microsatellite genotypes of the five colonies of *Ae. aegypti* in this study to previously reported populations. Populations 40–44 represent the following colonies: GH 98, GH 23, GH 115, GH 106, and AEG HCM, respectively. Previously reported populations [26] on the left represent those of non-African lineage, while those on the right are of African lineage: Argentina = 1, Australia = 2, Brazil = 3, Cameroon = 4, Colombia = 5, Costa Rica = 6, Dominican Republic = 7, French Polynesia = 8, Gabon = 9, Grenada = 10, Guinea-Bissau = 11, Indonesia = 13, Kenya = 14, Madeira = 15, Mauritius = 16, Mexico = 17, Hawaii = 25, Pakistan = 26, Puerto Rico = 27, Saudi Arabia = 28, Senegal = 29, South Africa = 30, Sri Lanka = 31, Thailand = 32, The Philippines = 33, Trinidad = 34, Uganda = 35, United States of America = 36, Venezuela = 37, Vietnam = 38

In confirming the lineages of the five *Ae. aegypti* colonies examined, 12 microsatellite loci were independently genotyped in each population by discriminant analysis of principal components (DAPC) and compared to previously reported genotypes of *Ae. aegypti* populations collected globally [25]. The comparative analysis of genotypic information revealed two distinct *Ae. aegypti* populations, those distributed in sub-Saharan Africa and classically designated as *Ae. aegypti formosus* (Fig. 1, blue and orange, right-hand side), and those distributed in the tropical and sub-tropical areas outside of Africa, designated *Ae. aegypti aegypti* (Fig. 1, gray and yellow, left-hand side) [11]. The four Ghanaian colonies (populations 40–43) clustered with mosquitoes of African origin. Although the control Vietnamese colony (population 44) did not cluster with previously reported field strains from Vietnam (population 38), possibly due to inbreeding under laboratory conditions [30], it clustered with *Ae. aegypti aegypti* populations with lineages outside Africa falling between field *Ae. aegypti aegypti* populations from Argentina and Madeira (populations 1 and 15, respectively) (Fig. 1).

### Susceptibility to DENV infection

**Fig. 2:**
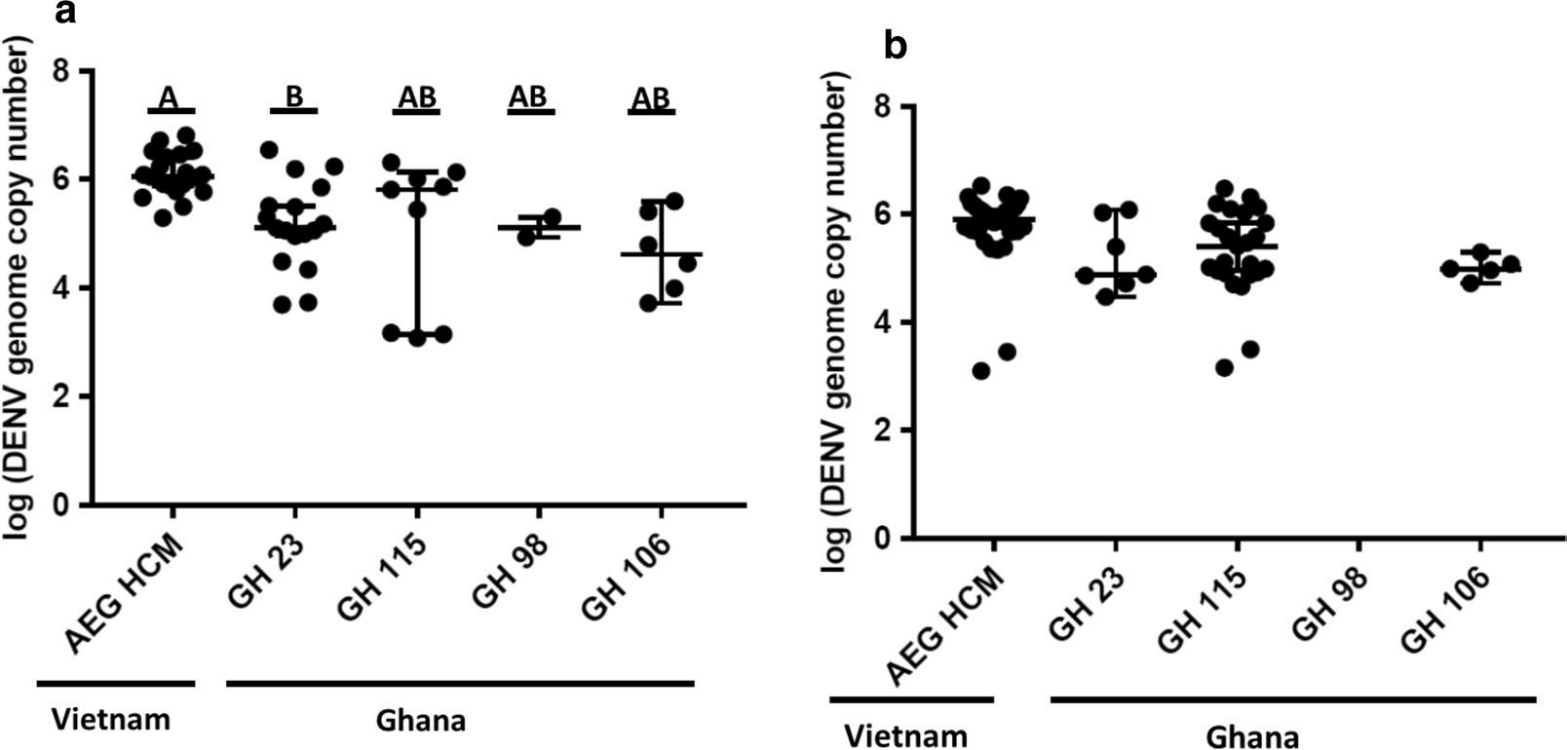
DENV-1 genome copies in infected thorax/abdomen. **a** Indicates DENV-1 genome copies, in the thorax/abdomen of *Ae. aegypti* colonies, at 7 dpi and 14dpi (**b**). Errors bars represent median with 95% confidence intervals. Dot plots with the same letters are not significantly different. Letters from A–B, above each column, represent a decrease in the average genome copies of DENV-1. Statistical significance, *P* < 0.05 was determined by the Kruskal–Wallis test with Bonferroni correction

**Fig. 3:**
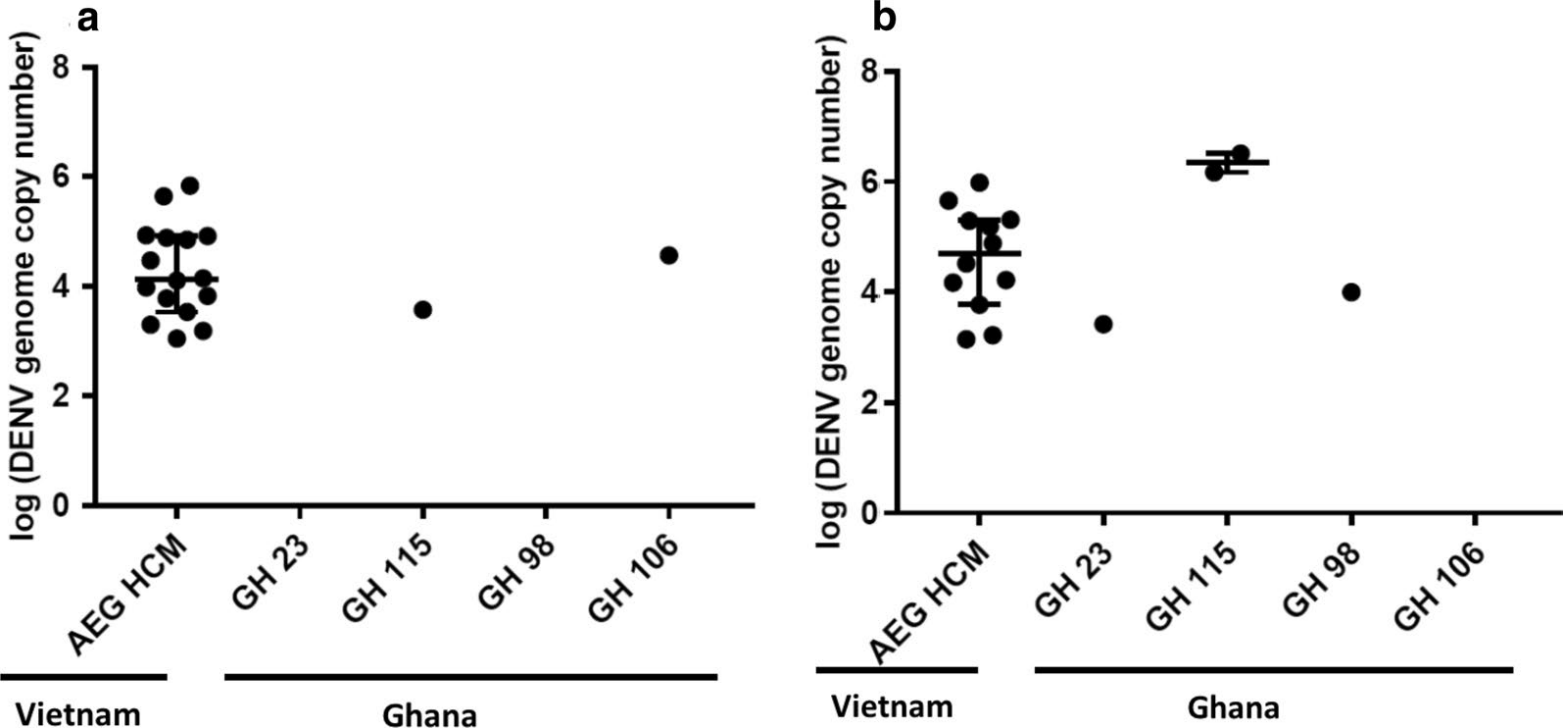
DENV-2 genome copies in infected thorax/abdomen. **a** DENV genome copies in the thorax/abdomen of *Ae. aegypti* colonies at 7 dpi and 14 dpi (**b**). Errors bars represent median with 95% confidence intervals. Statistical analysis was performed by Kruskal–Wallis test with Bonferroni correction

In determining the susceptibility of the *Ae. aegypti* colonies to DENV-1 and DENV-2 infection, the thorax/abdomen of blood-fed mosquitoes were screened at both 7 and 14 days post-infection (dpi). Colonies collected from the urban and sub-urban parts of Ghana, GH 115 and GH 23, respectively, were more susceptible to DENV-1 infection compared to DENV-2. GH 115 exhibited a 45% infection rate on exposure to DENV-1 but only 4% following exposure to DENV-2, while GH 23 had infection rates of ~ 41% and ~ 3% upon exposure to DENV-1 and DENV-2, respectively. GH 98 and GH 106, from rural Ghana, on the other hand were refractory to both serotypes of DENV (Table 3). The control group AEG HCM was significantly more susceptible to DENV infection as compared to the Ghanaian mosquitoes with a 75% and ~ 27% infection rate on exposure to DENV-1 and DENV-2, respectively. In terms of the distribution of susceptible mosquitoes between the two collection times, the proportion of mosquitoes infected with DENV-1 was significantly higher at 7 dpi than at 14 dpi only for colony GH 23 (Table 3). Number of genome copies of both serotypes in the thorax/abdomen of susceptible mosquitoes was however not significantly different among the Ghanaian colonies (Figs. 2 and 3). Of note, AEG HCM had significantly higher number of genome copies of DENV-1 than GH 23 and GH 106 (Fig. 2).

**Table 3:** Summary of results of *Ae. aegypti* colonies’ exposure to DENV

		7 dpi			14 dpi			Combined		
Virus	Mosquito colony	Infection rate (%)	Dissemination rate (%)	Transmission rate (%)	Infection rate (%)	Dissemination rate (%)	Transmission rate (%)	Infection rate (%)	Dissemination rate (%)	Transmission rate (%)
DENV-1	AEG HCM	75.9 (22/29)a	90.9 (20/22)a	0 (0/20)	74.5 (35/47)a	88.6 (31/35)a	51.6 (16/31)	75 (57/76)a	89.5 (51/57)a	31.4 (16/51)
	GH 23	68 (17/25)a	11.8 (2/17)b	0 (0/2)	21.2 (7/33)bc	71.4 (5/7)a	20 (1/5)	41.4 (24/58)b	29.2 (7/24)bc	14.3 (1/7)
	GH 115	39.1 (9/23)ab	22.2 (2/9)b	0 (0/2)	48.1 (25/52)ab	91.7 (23/25)a	34.8 (8/23)	45.3 (34/75)b	73.5 (25/34)ac	32 (8/25)
	GH 98	6.7 (2/30)b	0 (0/2)b	N/A	0 (0/35)c	N/A	N/A	3.1 (2/65)c	0 (0/2)bc	N/A
	GH 106	15.4 (6/39)b	16.7 (1/6)b	0 (0/1)	11.6 (5/43)c	40 (2/5)a	0 (0/2)	13.4 (11/82)c	27.3 (3/11)bc	0 (0/3)
DENV-2	AEG HCM	30.2 (16/53)a	12.5 (2/16)	0 (0/2)	24 (12/50)a	16.7 (2/12)	0 (0/2)	27.2 (28/103)a	14.3 (4/28)	0 (0/4)
	GH 23	0 (0/21)ab	N/A	N/A	5.6 (1/18)ab	0 (0/1)	N/A	2.6 (1/39)ab	0 (0/1)	N/A
	GH 115	2.5 (1/40)b	0 (0/1)	N/A	5.7 (2/35)ab	0 (0/2)	N/A	4 (3/75)b	0 (0/3)	N/A
	GH 98	0 (0/42)b	N/A	N/A	2.3 (1/44)ab	0(0/1)	N/A	1.2 (1/86)b	0 (0/1)	N/A
	GH 106	2.6 (1/38)b	0 (0/1)	N/A	0 (0/41)b	N/A	N/A	1.3 (1/79)b	0 (0/1)	N/A

This table compares the infection, dissemination, and transmission rates of all five mosquito colonies exposed to DENV-1 and DENV-2. The data show the significant superiority of AEG HCM as a vector of DENV relative to all four colonies from Ghana, particularly in terms of susceptibility to DENV infection and dissemination. Numbers in parenthesis show the ratio of positive females to the total number of females screened. Colonies denoted with the same lowercase letter for a given parameter are not significantly different. Statistical significance (*P* < 0.005) was determined by the Chi squared test with Bonferroni correction

### Dissemination of DENV in infected mosquitoes

**Fig. 4:**
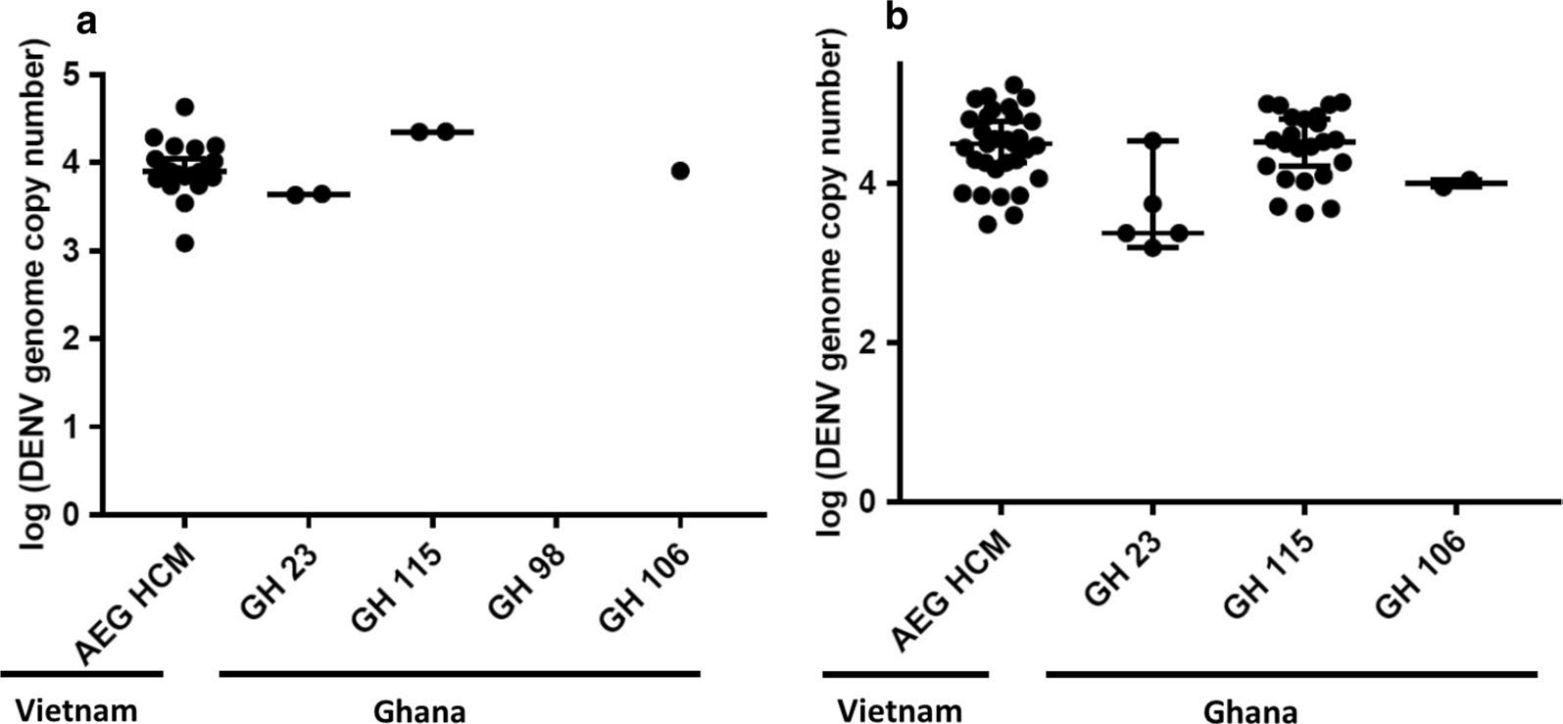
DENV-1 genome copies in the head/wings/legs of mosquitoes with disseminated infection. **a** Average genome copies of DENV-1 in the head/wings/legs at 7 dpi and 14 dpi (**b**). Error bars represent the median with 95% confidence intervals. Statistical analysis was done by Kruskal–Wallis test with Bonferroni correction

**Fig. 5:**
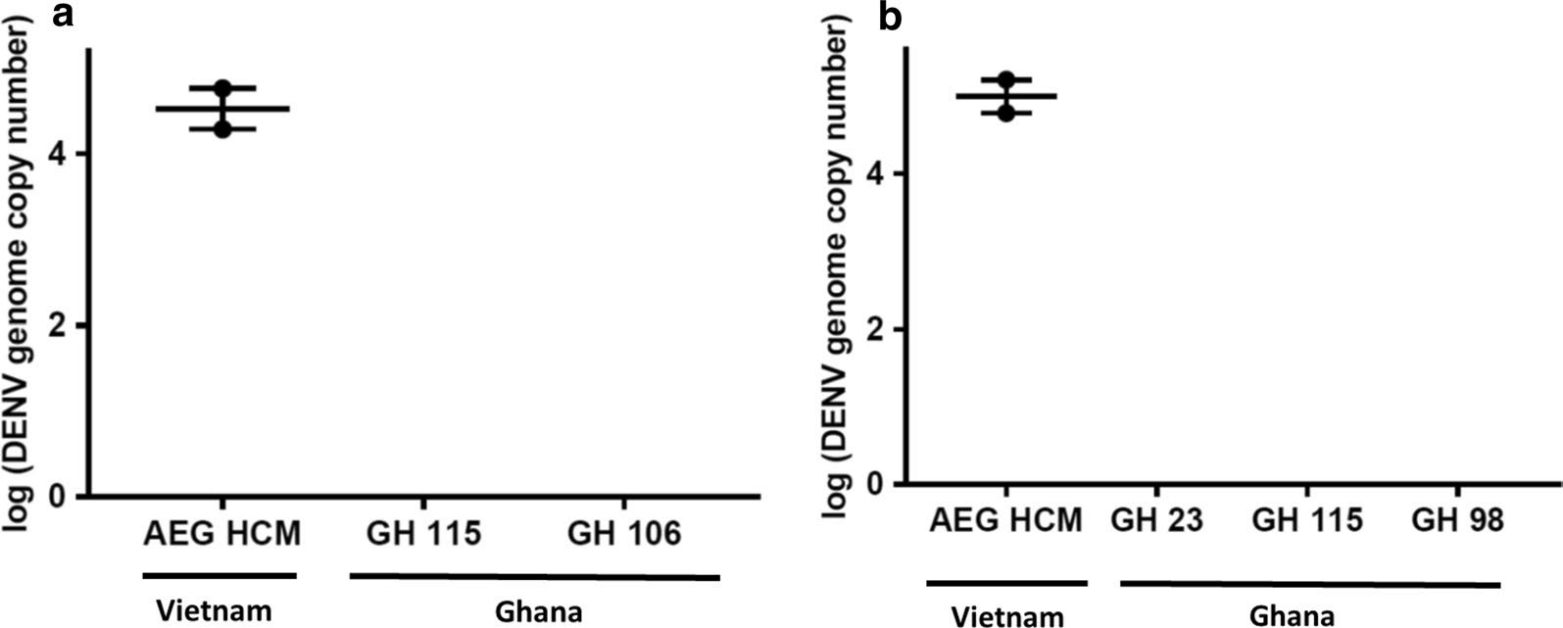
DENV-2 genome copies in the head/wings/legs of mosquitoes with disseminated infection. DENV-2 genome copies in the head/wings/legs at 7 dpi (**a**) and 14 dpi (**b**). Dissemination occurred only in AEG HCM. Error bars represent median with 95% confidence intervals

The head/wings/legs of all infected mosquitoes were screened for DENV to determine whether DENV-1 and DENV-2 disseminated from the midgut to various body parts. Consistent with observations on susceptibility, dissemination within the AEG HCM strain was significantly higher than of any of the Ghanaian mosquitoes and was higher for DENV-1 (~ 90%) compared to DENV-2 (~ 14%) (Table 3). Furthermore, dissemination within Ghanaian colonies peaked after 7 dpi, while peak dissemination occurred within 7 dpi in the AEG HCM colony. Among the Ghanaian colonies however, GH 115 was the most susceptible to dissemination (mean value ~ 74%; value at 14 dpi ~ 92%) although there was no significant difference in the number of genome copies between mosquitoes susceptible to disseminated infection. GH 23 and GH 106, on the other hand, had mean dissemination values of ~ 29% and ~ 27%, respectively. There was no DENV-1 dissemination detected in GH 98, and DENV-2 did not disseminate in any Ghanaian mosquito studied (Figs. 4 and 5; Table 3).

### Transmission potential of the *Ae. aegypti* colonies

**Fig. 6:**
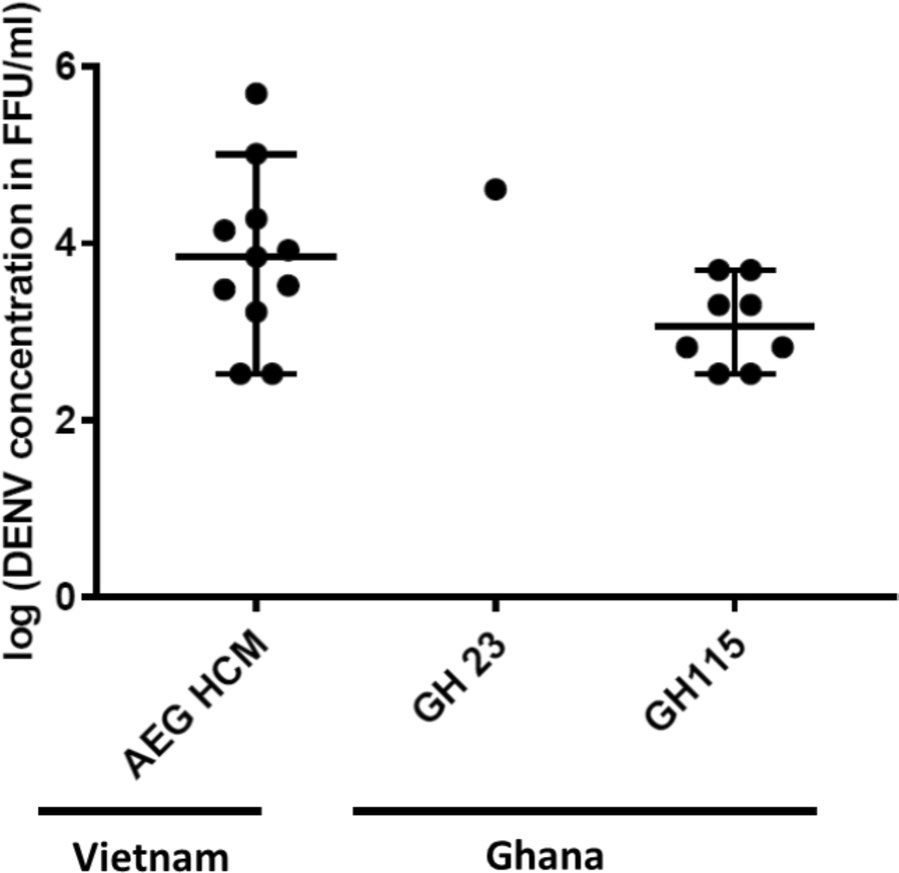
Concentration of DENV-1 in infectious mosquito saliva. Error bars represent 95% confidence intervals. Statistical analysis was performed by Kruskal–Wallis test with Bonferroni correction

To determine the potential to transmit DENV, the saliva of mosquitoes that exhibited virus dissemination was screened. The minimum length of time required to detect DENV-1 in the saliva of any of the five colonies was over 7 dpi (Table 3). By 14 dpi, however, ~ 52% of AEG HCM, ~ 20% of GH 23, ~ 35% of GH 115, and 0% of GH 106 mosquitoes were infectious, accounting for ~ 22%, ~ 2%, ~ 11%, and 0%, respectively, of the total number of mosquitoes per colony exposed to DENV-1 in this study. The only colony with DENV-2 dissemination was AEG HCM, and by 14 dpi this colony had not become infective (Table 3). In terms of viral load, the AEG HCM strain recorded the highest viral titer in the saliva, but there was no significant difference in the average viral titer between the colonies with infectious saliva (Fig. 6).

## Discussion

*Aedes aegypti* plays the crucial role of primary vector in DENV transmission. The continuous spread of this vector into new areas coupled with its endophilic nature and high affinity for humans increases the potential of DENV transmission in all areas where the mosquito is present [31]. This notwithstanding, DENV is not endemic in all areas where the vector is present and the intensity of outbreaks differs significantly geographically, which has led to increased interest in determining the impact of vector competence in the transmission of DENV. Furthermore, recent reports on *Ae. aegypti* from West Africa, including the forest dwellers classically designated as *Ae. aegypti formosus,* have established the competence of West African mosquitoes in Senegal as DENV vectors [13]. This study therefore sought to determine the vector competence of *Ae. aegypti* mosquitoes collected from four different locations in Ghana, where dengue is thought to be virtually absent, at least based on the available data. Due to confirmed endemicity of DENV in Vietnam (i.e. Ho Chi Minh City) and reports of Vietnamese *Ae. aegypti* competency [32–35]*,* a colony from this region was used as the control group. This study found *Ae. aegypti* from Ghana to have varying degrees of DENV-vector susceptibility and competence, with those from the South of Ghana relatively more competent.

The four aforementioned locations in Ghana were selected to allow for comparison of DENV transmission and outbreak risks in urban and semi-urban locations in the south (GH 115 and GH 23, respectively) to rural areas in the north (GH 98 and GH 106) [18]. In addition to performing infection experiments, the lineages of all five *Ae. aegypti* colonies were confirmed by DAPC analysis [26]. Although the mosquitoes collected in Ghana phylogenetically clustered with those classically designated as *Ae. aegypti formosus*, their breeding sites and habitats were not the same as those predicted for *Ae. aegypti formosus*. These results are consistent with others from the West African sub-region that highlight difficulties in subspecies identification using the traditionally accepted distinctions [13]. Furthermore, this study exposed the mosquito colonies to two serotypes of DENV, including DENV-2 isolated from a patient in Ghana [2]. Determining the vector competence of Ghanaian *Ae. aegypti* in transmitting the DENV-2 strain in this study was important for two reasons: this serotype reportedly has a high potential of emergence [13], and this is possibly the strain in circulation in Ghana, if any.

Prior to performing infections, the optimal conditions for viral infectivity in mosquitoes were determined. Consistent with previous reports, a virus concentration of 1–5 × 10^6^ focus forming units (FFU)/ml and the avoidance of freeze and thaw were necessary for optimal infection [28, 31]. Harvested DENV-infected cell culture supernatant was therefore stored on ice until used. On exposure of the mosquitoes to DENV, three main parameters were investigated: infection rate, dissemination rate, and transmission rate; thus, by extension the extrinsic incubation period (EIP) was also determined.

The infection rate was defined as the proportion of exposed mosquitoes that exhibited susceptibility to DENV infection. The susceptibility of a mosquito colony to DENV infection is dependent on the ability of the virus to evade the midgut infection barrier (MIB). The MIB prevents the virus from gaining entry to the midgut cells through absence of receptors on the surface of the epithelial cells and the existence of the peritrophic matrix that some pathogens are unable to penetrate [36, 37]. This study found all susceptible mosquito colonies to have higher susceptibility to DENV-1 than to DENV-2, despite the fact that the DENV-2 strain used was isolated from a patient in Ghana. This observation is not completely unexpected as there have been several reports on the varying degrees of mosquito colony susceptibility to different serotypes, as well as strains of a given serotype [1, 38]. With regard to differences in susceptibility between the examined colonies, however, AEG HCM was significantly more susceptible to DENV infection (both serotypes) than the most susceptible Ghanaian mosquito colonies (GH 23 and GH 115). This may imply that the MIB in these Ghanaian mosquitoes more efficiently prevented DENV colonization of the midgut. The colonies collected from the southern urban and semi-urban areas of Ghana, GH 23 and GH 115, were significantly more susceptible to DENV infection than those from the rural north. Lastly, in this study, the randomized distribution of mosquitoes into groups following DENV exposure may have resulted in the uneven distribution of susceptible mosquitoes between collection points observed in the GH 23 colony exposed to DENV-1.

The next barrier a virus has to overcome after establishing infection and replicating in the mosquito midgut is the midgut escape barrier (MEB), which prevents the spread of the virus to the hemolymph or secondary organs [31, 37]. Successful progression into the hemocoel and subsequent evasion of a mosquito’s innate immunity results in the dissemination of the virus to other organs. In this study, both DENV serotypes were capable of disseminating to other organs of AEG HCM mosquitoes within 7 dpi. However, this period was not sufficient for significant dissemination to occur in any of the Ghanaian colonies, with DENV-2 requiring more than 14 days to potentially disseminate in these mosquitoes. Again, the colonies collected in the south of Ghana had a significantly higher dissemination rate than those collected in the north.

Last but not least, the virus must overcome the salivary gland infection and escape barriers (SGIB and SGEB) for successful transmission into a human host. The SGIB restricts DENV infection of the salivary gland by shielding the cellular entry cells with the basal lamina, which is typically more efficient when the viral titer is low [37]. If the virus successfully enters the salivary gland, it goes through cycles of replication to increase the viral titer and enhance the chances of successful transmission. Replication can be hindered by immune reactions within the mosquito [39, 40]. The EIP, which is the period between mosquito infection and transmission of DENV, is typically 7–14 days [31] but may extend to about 33 days [41]. In this study, AEG HCM was relatively the most proficient at transmitting DENV-1, as this colony exhibited a greater proportion of infectious individuals and harbored an average viral load a hundred times greater than that of GH 23 or GH 115. The reduced viral load observed in GH 23 and GH 115 and the absence of infectious individuals in GH 98 and GH 106 could be due to the SGIB, SGEB, and immune reactions within the salivary glands. The DENV-2 strain used in this study, on the other hand, appeared to have an EIP ≥ 14 days in AEG HCM, which was the only colony to disseminate the virus to other body parts from the midgut. While this increased EIP may reduce transmission efficiency, it would be interesting to determine the exact EIP for this DENV-2 strain and the reasons underlying its apparently reduced transmission efficiency.

The results of this study clearly show the colonies from Ghana to be highly refractory to the DENV-2 isolated from a patient in that country. Although this may be an indication as to why this strain has not emerged and caused a large-scale outbreak in Ghana despite proof of its presence and possible circulation, the sufficient competency exhibited by mosquitoes collected in the south, GH 23 and GH 115, in transmitting DENV-1 (Table 1), should raise concerns about the possible transmission of other DENV strains upon exposure. This indicates a need to establish periodic monitoring of DENV transmission status in Ghana, possibly by employing non-invasive entomological tools such as mosquito screening for DENV and other arboviruses, particularly in the south. Furthermore, taking into account the ease of movement within the West African Sub region especially, it would be important to put measures in place to prevent or ensure early detection of any strain that could potentially cause an outbreak, since at least one of the 4 known cases in Ghana was confirmed to have been imported [19]. Lastly, with confirmed interbreeding between *Ae. aegypti* of African lineage and those of non-African lineage [11, 13], it will be equally important to determine the effect of crossbreeding on vector competence while also checking for the invasion of vectors of non-African lineage, particularly in communities close to ports of entry in the south of Ghana.

## Conclusions

We found Ghanaian *Ae. aegypti* to be refractory to the DENV-2 strain isolated from a patient in Ghana but showed different levels of susceptibility to the DENV-1 strain isolated from a patient in Japan. These findings therefore highlight the need for continuous surveillance for potential outbreaks while giving insight into the possible risk of outbreaks, particularly in the urban areas in the south of Ghana.

## Data Availability

The datasets used and/or analyzed during the current study are available from the corresponding author on reasonable request.

## Abbreviations

DENV: Dengue virus
MIB: Midgut infection barrier
MEB: Midgut escape barrier
SGIB: Salivary gland infection barrier
SGEB: Salivary gland escape barrier
EIP: Extrinsic incubation period
DAPC: Discriminant analysis of principal components
